# Supplementary material for: Content-rich biological network constructed by mining PubMed abstracts
Source: BMC Bioinformatics. 2004 Oct 8;5:147. doi: 10.1186/1471-2105-5-147 (PMC528731; doi:10.1186/1471-2105-5-147)
Supplement: Additional File 2 — The original results of the above study (non-essential files are deleted to keep the file size under the limit set by BMC bioinformatics). [file 1471-2105-5-147-S2.bz2 › chilibotAdditionalFile2/dip10/155ID8822197E554/html/RB1_CDK6.html]

 


 **RB1** and **CDK6** 
  
Found 151 abstracts in PubMed, retrieved 10.  
 

 What does Google say? 
 PDF only 
| .edu only 

---

**Interactive relationship** (e.g. stimulation, inhibition, etc)

**Inhibitory relationship**- At the molecular level, the tetramer inhibited cyclin dependent kinase CDK 6  [ **CDK6** ]  expression and activity, induced an increase in the level of p27 kip1, and reduced the level of retinoblastoma  [ **RB1** ]  protein expression.  Ref: 12874002 Cancer ResCancer Res,
**Neutral relationship**- D type cyclins bind to and activate the cyclin dependent kinases Cdk4 and  **Cdk6** , which in turn phosphorylate their downstream target, the retinoblastoma  [ **RB1** ]  protein Rb.  Ref: 12459251 Gene, 2002

**Non-interactive relationship** (e.g. studied together, co-existance, homology, etc.)

- Components upstream of the retinoblastoma  [ **RB1** ]  tumour suppressor pRB, namely the INK4 family of cyclin dependent kinase CDK inhibitors, the D type cyclins, their partner kinases CDK4  **CDK6** , and pRB as their critical substrate, are differentially targeted in diverse types of cancer.  Ref: 12754735 J Pathol< MedlineTA>J Pathol, 2002
